# Effect of fasting status on Alzheimer's disease blood biomarkers

**DOI:** 10.1002/alz70856_102762

**Published:** 2025-12-24

**Authors:** Bethany Schuder, Daniel Figdore, Joshua A Bornhorst, Alicia Algeciras‐Schimnich

**Affiliations:** ^1^ Mayo Clinic, Rochester, MN, USA

## Abstract

**Background:**

Alzheimer's disease (AD) blood biomarkers (BBMs) are increasingly used in clinical trials and clinical practice. However, widespread adoption of BBMs requires further understanding of factors that might influence the interpretation of these biomarkers which would allow for the development of standardized sample collection protocols. For example, studies of the effect of fasting on AD BBM concentrations have yielded inconsistent findings. In this study, we evaluated plasma concentrations of amyloid‐beta(Aβ) 42, Aβ40, phosphorylated Tau (*p*‐tau) 181, *p*‐tau217, and neurofilament light chain (NfL) in cognitively normal individuals under fasting and non‐fasting conditions.

**Method:**

EDTA‐plasma was collected from healthy donors (*n* = 14; age >55yrs old) two weeks apart, at the same time of day. The first sample collection was under fasting conditions in which donors did not eat or drink (except water) for at least 12 hours prior to collection. The second sample collection was under non‐fasting conditions in which donors were instructed to eat a meal in the morning, approximately half an hour prior to collection. Plasma Aβ42, Aβ40, *p*‐tau181, *p*‐tau217, and NfL concentrations were measured on the Fujirebio Lumipulse G1200 analyzer. Wilcoxon signed‐rank test was used to compare values between the fasting and non‐fasting results. Spearman's rank correlation was used to assess biomarker correlation between fasting status.

**Result:**

Moderate to strong correlation was observed between the fasting and non‐fasting measurements: *p*‐tau217 (r_s_: 0.86), *p*‐tau181 (r_s_: 0.89), NfL (r_s_: 0.94), *p*‐tau217/Aβ42 (r_s_: 0.80), Aβ42/40 (r_s_: 0.66), Aβ42 (r_s_: 0.77), and Aβ40 (r_s_: 0.92). Average percent difference between the 2 conditions was: *p*‐tau217 27%, *p*‐tau181 6%, NfL 9%, *p*‐tau217/ Aβ42 25%, Aβ42/40 4%, Aβ42 2%, and Aβ40 ‐6%. Statistically significant differences (*p* ≤ 0.05) between fasting and non‐fasting measurements were observed for *p*‐tau217, NfL, *p*‐tau217/Aβ42, Aβ42/Aβ40, and Aβ42; but not for Aβ40 or *p*‐tau181.

**Conclusion:**

This data suggests that for some AD BBMs, there may be statistically significant differences in concentrations of samples collected in fasting versus non‐fasting conditions. However, further studies are needed to determine if the observed differences may affect the clinical interpretation of these BBMs in individuals with amyloid related pathology.

